# Supplementary material for: Noncanonical roles of ATG5 and membrane atg8ylation in retromer assembly and function
Source: bioRxiv. 2024 Oct 14:2024.07.10.602886. Originally published 2024 Jul 12. Preprint. [Version 2] doi: 10.1101/2024.07.10.602886 (PMC11257513; doi:10.1101/2024.07.10.602886)
Supplement: Supplement 1 [file NIHPP2024.07.10.602886v2-supplement-1.pdf]

bioRxiv preprint doi: https://doi.org/10.1101/2024.07.10.602886; this version posted October 14, 2024. The copyright holder for this preprint (which was not certified by peer review) is the author/funder, who has granted bioRxiv a license to display the preprint in perpetuity. It is made available under aCC-BY 4.0 International license.

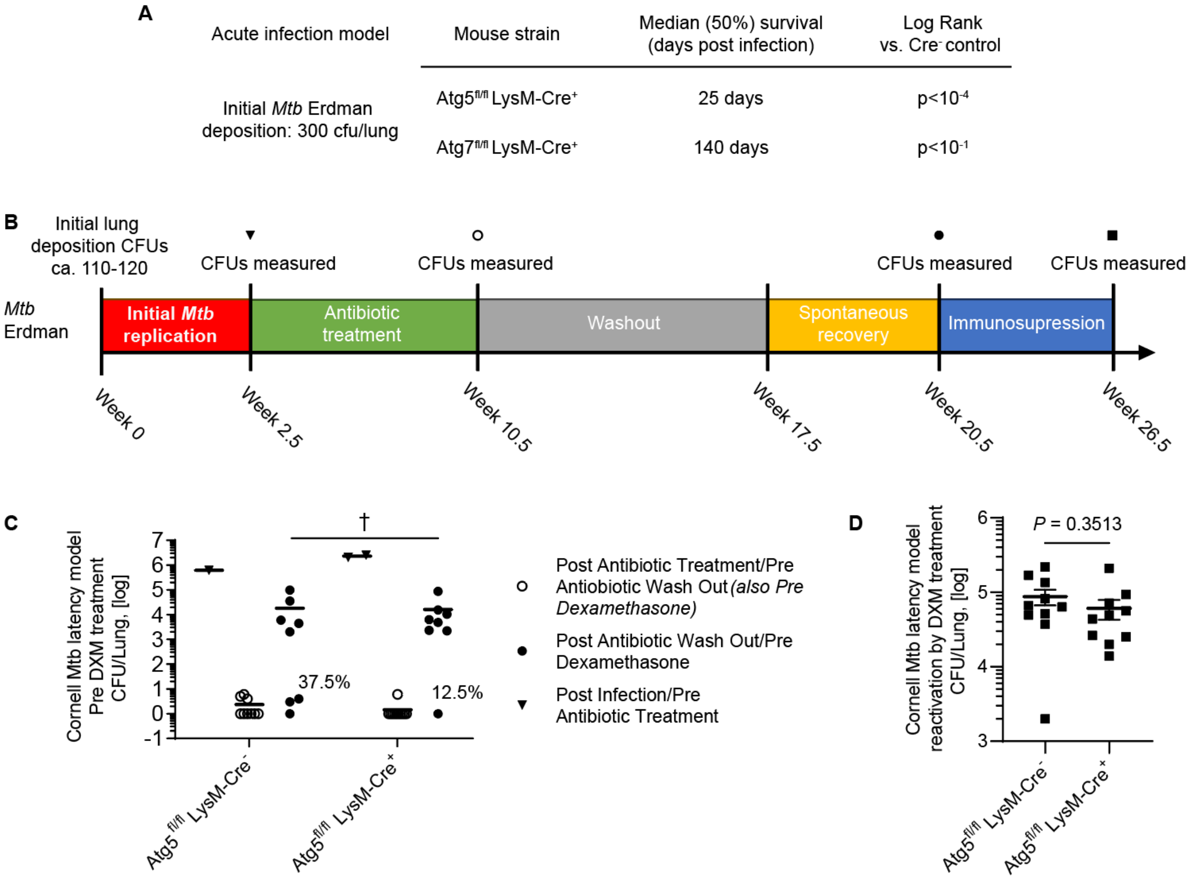

Supplementary Figure S1. Cornell model of *M. tuberculosis* latent infection in mice and effects of Atg5 loss in myeloid lineage on disease reactivation. A. Summary of mice mortality data in acute infection model of *Mtb* infection (aerosol) based on survival curves in ref. 35. **B.** Details and timeline of the Cornell latency model experiments (see narrative in Methods). **C.** Effects of Atg5 loss on spontaneous reactivation of *M. tuberculosis* infection in Atg5$^{fl/fl}$ LysM-Cre^{+} mice (loss of Atg5 in myeloid lineage) vs. Atg5$^{fl/fl}$ LysM-Cre^{-} (control) mice. Mice were infected with an aerosol of *M. tuberculosis* (initial deposition, 100-120 CFUs per lung). After a period of 2.5 weeks, initial bacterial growth was assessed by determining lung CFUs (triangles), mice were treated PO with antibiotics (0.1g/L INH and 0.15g/L RIF in drinking water) for 8 weeks, bacterial clearance after chemotherapy assessed by determining lung CFUs (open circles), and remaining mice subjected to antibiotic washout for 7 weeks plus spontaneous reactivation period with no treatment of 3 weeks at which time the mice were sacrificed and lung CFUs determined by plating (filled circles). Data and statistics for spontaneous reactivation:

bioRxiv preprint doi: https://doi.org/10.1101/2024.07.10.602886; this version posted October 14, 2024. The copyright holder for this preprint (which was not certified by peer review) is the author/funder, who has granted bioRxiv a license to display the preprint in perpetuity. It is made available under aCC-BY 4.0 International license.

means, †p≥0.05, *t* test; n=8 mice per group. **D.** Cornell murine model of *M. tuberculosis* latent infection and dexamethasone (DXM) induced reactivation and effects of Atg5 loss in myeloid lineage of Atg5$^{fl/fl}$ LysM-Cre^{+} mice (vs. Atg5$^{fl/fl}$ LysM-Cre^{-} control mice). Mice were infected with *M. tuberculosis* aerosols (initial lung deposition 100-120 CFUs), bacteria allowed to replicate in vivo, mice subjected to antibiotic regimen, followed by antibiotic washout period, after which immunosuppression with DXM was carried out to reactivate infection/bacterial replication (details in Methods). Data, CFU's per mouse lungs (means ± SE, *t* test, n=10 mice per group).

bioRxiv preprint doi: https://doi.org/10.1101/2024.07.10.602886; this version posted October 14, 2024. The copyright holder for this preprint (which was not certified by peer review) is the author/funder, who has granted bioRxiv a license to display the preprint in perpetuity. It is made available under aCC-BY 4.0 International license.

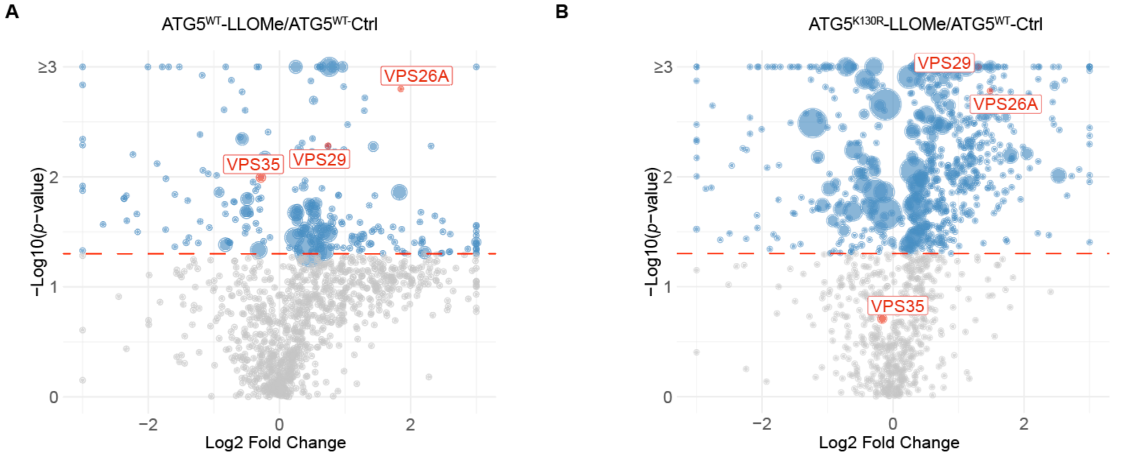

C

Protein name	% of control	
	ATG5^{WT} LLOMe/Ctrl	ATG5^{K130R} LLOMe/ ATG5^{WT}Ctrl
VPS26A	360%	279%
VPS29	167%	246%
VPS35	82%	89%
VPS45	81%	72%
VPS51	50%	56%

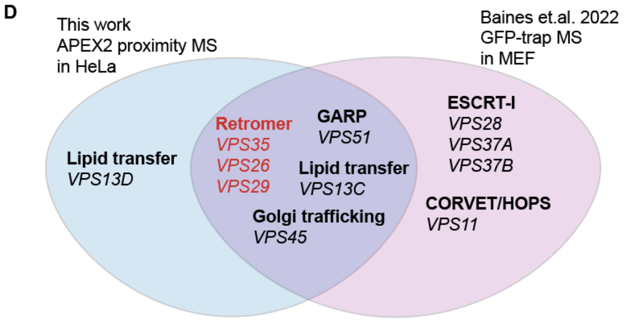

E

APEX2-SGALS1

LLOMe/Ctrl	
Retromer subunit	% of control
VPS26A	67%
VPS29	102%
VPS35	101%

Supplementary Figure S2. ATG5 interactome analysis. A. Volcano plot of proximity biotinylation LC-MS/MS interactome comparing FlpIn-HeLa$^{APEX2\text{-}ATG5\text{-}WT}$ treated with or without 2 mM LLOMe for 30 min. Diameter of symbols reflects relative number of unique peptides identified. **B**. Volcano plot of proximity biotinylation LC-MS/MS interactome comparing FlpIn-HeLa$^{APEX2\text{-}ATG5\text{-}K130R}$ treated with 2 mM LLOMe for 30 min and FlpIn-HeLa$^{APEX2\text{-}ATG5\text{-}WT}$ without LLOMe treatment. **C**. VPS proteins in the MS DIA data. **D.** VPS proteins identified here compared to VPS proteins in Ref. 86. **E.** Table: Control MS data (APEX2-SGALS1) for comparison with data in panel C; note no increase in retromer subunits with LLOMe treatment in the APEX1-SGALS1 dataset.

bioRxiv preprint doi: https://doi.org/10.1101/2024.07.10.602886; this version posted October 14, 2024. The copyright holder for this preprint (which was not certified by peer review) is the author/funder, who has granted bioRxiv a license to display the preprint in perpetuity. It is made available under aCC-BY 4.0 International license.

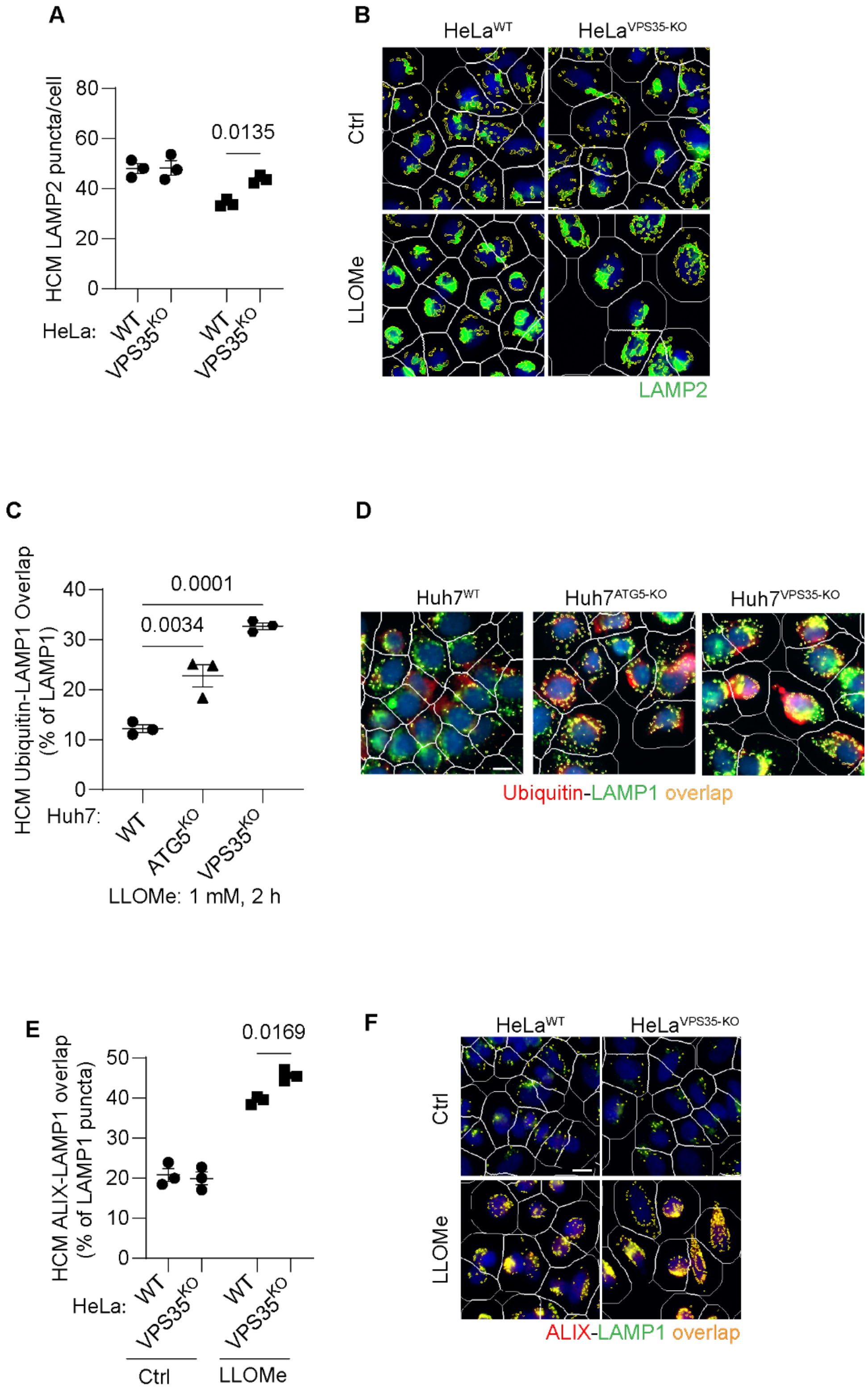

Supplementary Figure S3. Retromer affects a subset of responses to lysosomal damage. A,B. HCM imaging and quantification of LC3 (puncta/cell of immunofluorescently stained endogenous LC3 profiles) in HeLa^{WT}, and HeLa$^{VPS35\text{-}KO}$ cells

bioRxiv preprint doi: https://doi.org/10.1101/2024.07.10.602886; this version posted October 14, 2024. The copyright holder for this preprint (which was not certified by peer review) is the author/funder, who has granted bioRxiv a license to display the preprint in perpetuity. It is made available under aCC-BY 4.0 International license.

in response to lysosomal damage by LLOMe. Data, means ± SE (n=3), one-way ANOVA with Tukey's multiple comparisons. **C,D**. HCM analysis of ubiquitin (immunofluorescence; FK2 antibody) response to lysosomal damage (LLOMe; 1mM, 2 h) in Huh7^{WT}, Huh7$^{ATG5-KO}$, and Huh7$^{VPS35-KO}$ cells. Yellow profiles, colocalization of ubiquitin and LAMP1. Data, means ± SE (n=3), one-way ANOVA with Tukey's multiple comparisons. **E,F.** HCM quantification of ALIX localization to endolysosomal compartments (% of LAMP1 profiles positive for ALIX immunostaining) in HeLa^{WT} and HeLa$^{VPS35-KO}$ cells following lysosomal damage. Yellow profiles, colocalization of ALIX and LAMP1. Data, means ± SE (n=3); two-way ANOVA with Tukey's multiple comparisons. HCM images in all relevant panels, examples from a bank of unbiased operator-independent machine-collected and algorithm-processed fields containing a minimum of 500 primary objects/cells per well (5 wells minimum per 96-well plate; 3 plates minimum), per cell line/condition.

bioRxiv preprint doi: https://doi.org/10.1101/2024.07.10.602886; this version posted October 14, 2024. The copyright holder for this preprint (which was not certified by peer review) is the author/funder, who has granted bioRxiv a license to display the preprint in perpetuity. It is made available under aCC-BY 4.0 International license.

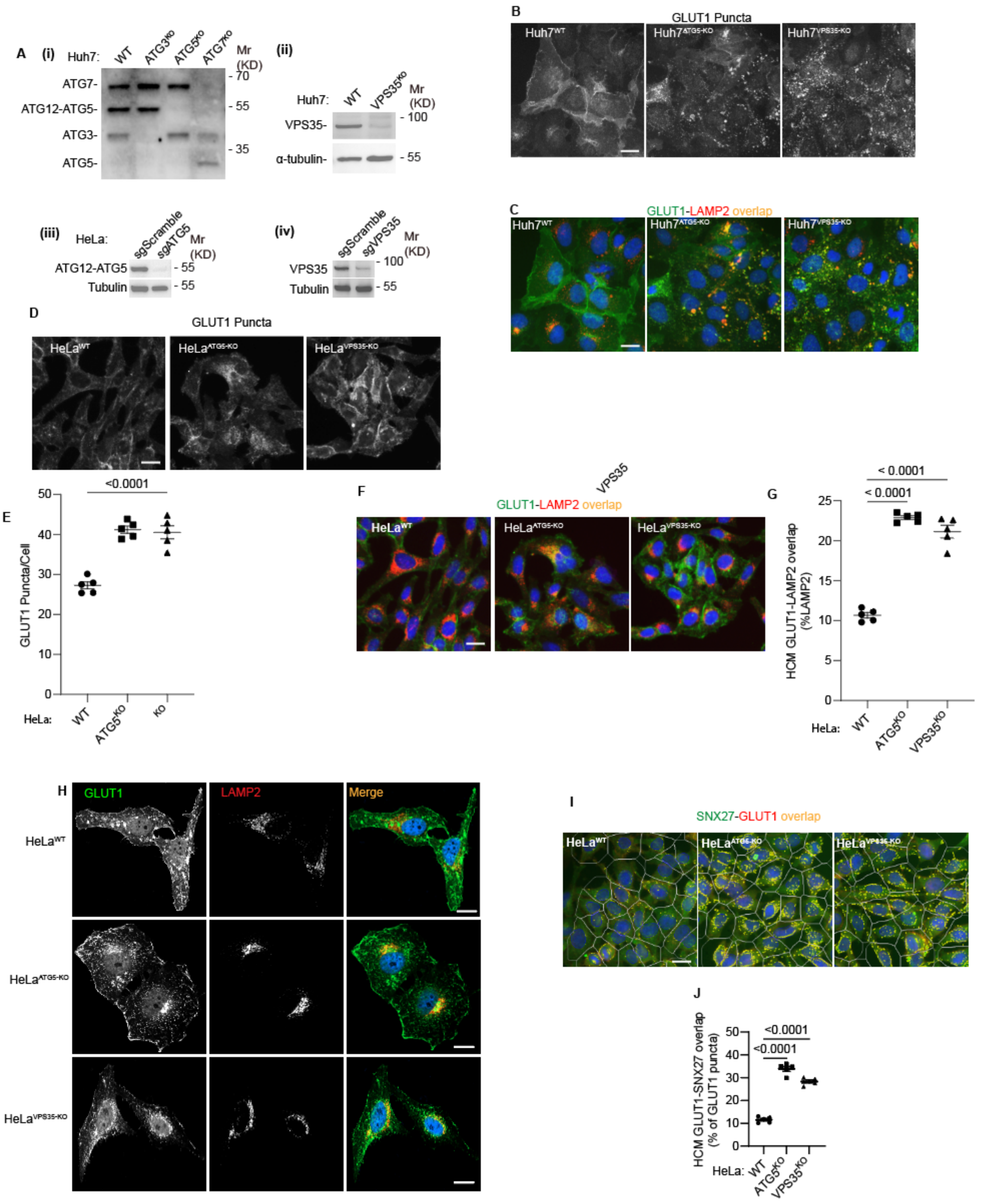

Supplementary Figure S4. Membrane atg8ylation regulates retromer function. A. Immunoblots of CRISPR KOs: (i) ATG3, ATG5, and ATG7 in Huh7 cells; (ii) VPS35 in Huh7 cells; (iii) ATG5 in HeLa cells; and (iv) VPS35 in Hela cells. **B,C.** HCM images

bioRxiv preprint doi: https://doi.org/10.1101/2024.07.10.602886; this version posted October 14, 2024. The copyright holder for this preprint (which was not certified by peer review) is the author/funder, who has granted bioRxiv a license to display the preprint in perpetuity. It is made available under aCC-BY 4.0 International license.

(example form a bank of unbiased operator-independent machine collected and processed images containing a minimum of 500 primary objects/cells) of GLUT1 (immunostaining of endogenous protein) puncta/cell (B) and GLUT1 colocalization with LAMP2 in Huh7^{WT}, Huh7$^{ATG5\text{-}KO}$, and Huh7$^{VPS35\text{-}KO}$ cells (C). Scale bar, 20 μm. **D,E.** HCM quantification of GLUT1 (immunostaining of endogenous protein) puncta/cell in HeLa^{WT}, HeLa$^{ATG5\text{-}KO}$, and HeLa$^{VPS35\text{-}KO}$ cells. Scale bar, 20 μm. Data, means ± SE (n =5); one-way ANOVA with Tukey's multiple comparisons. **F,G.** HCM quantification of GLUT1 colocalization with LAMP2 (% of LAMP2 profiles positive for GLUT1; overlap area) in HeLa^{WT}, HeLa$^{ATG5\text{-}KO}$, and HeLa$^{VPS35\text{-}KO}$ cells. Scale bar, 20 μm. Data, means ± SE (n =5); one-way ANOVA with Tukey's multiple comparisons. **H.** Confocal images illustrating localization of GLUT1 and LAMP2 in HeLa^{WT}, HeLa$^{ATG5\text{-}KO}$, and HeLa$^{VPS35\text{-}KO}$ cells. Scale bar, 10 μm. **I,J.** HCM quantification of GLUT1-SNX27 overlap (% of SNX7 area positive for GLUT1) in HeLa^{WT}, HeLa$^{ATG5\text{-}KO}$, and HeLa$^{VPS35\text{-}KO}$. Scale bar, 20 μm. Data, means ± SE (n=4); one-way ANOVA with Tukey's multiple comparisons. HCM images in all relevant panels, examples from a bank of unbiased operator-independent machine-collected and algorithm-processed fields containing a minimum of 500 primary objects/cells per well (5 wells minimum per 96-well plate; 3 plates minimum), per cell line.

bioRxiv preprint doi: https://doi.org/10.1101/2024.07.10.602886; this version posted October 14, 2024. The copyright holder for this preprint (which was not certified by peer review) is the author/funder, who has granted bioRxiv a license to display the preprint in perpetuity. It is made available under aCC-BY 4.0 International license.

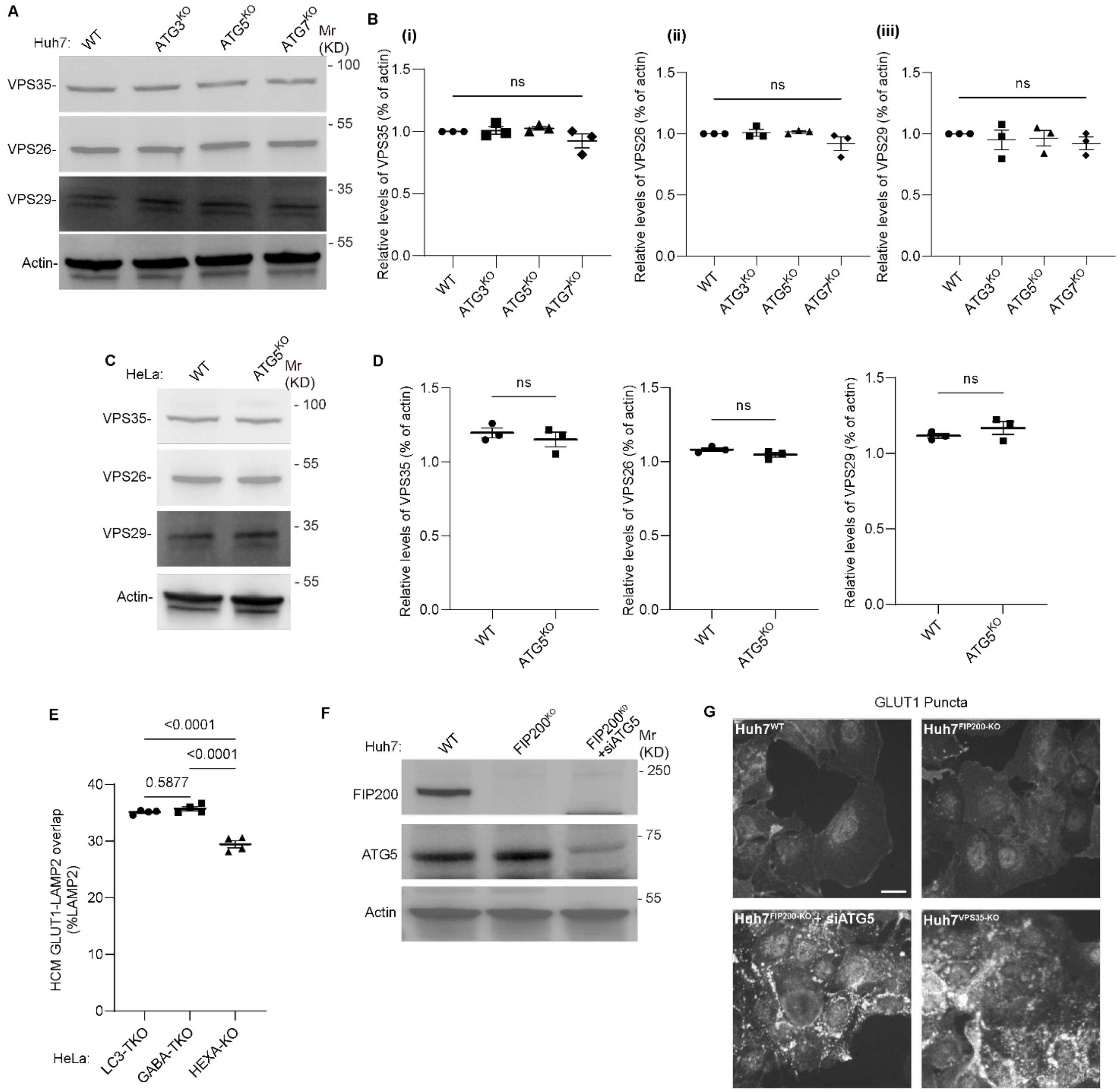

Supplementary Figure S5. ATG5 knockout has no effect on protein levels of retromer subunits. A,B. Immunoblot analysis (A) and quantification (B) of retromer complex proteins VPS35 (i), VPS26 (ii), and VPS29 (iii) from Huh7^{WT}, Huh7$^{ATG3-KO}$, Huh7$^{ATG5-KO}$, and Huh7$^{ATG7-KO}$ cells (total cell extract). **C,D.** Immunoblot analysis (C) and quantification (D) of retromer complex proteins VPS35 (i), VPS26 (ii), and VPS29 (iii) from HeLa^{WT}, and HeLa$^{ATG5-KO}$ cells (total cell extract). **E.** HCM quantification of GLUT1-LAMP2 colocalization in HeLa$^{LC3-TKO}$, HeLa$^{GABA-TKO}$, and HeLa$^{HEXA-KO}$ cells. Data, means

bioRxiv preprint doi: https://doi.org/10.1101/2024.07.10.602886; this version posted October 14, 2024. The copyright holder for this preprint (which was not certified by peer review) is the author/funder, who has granted bioRxiv a license to display the preprint in perpetuity. It is made available under aCC-BY 4.0 International license.

± SE (n =4), one-way ANOVA with Tukey's multiple comparisons; ns (not significant), $p > 0.05$. **F.** Immunoblot analysis showing the siRNA mediated knockdown of ATG5 in Huh7$^{FIP200\text{-}KO}$ cells. **G.** HCM images of GLUT1 puncta/cell in Huh7^{WT}, Huh7$^{FIP200\text{-}KO}$, Huh7$^{FIP200\text{-}KO+\ siATG5}$, and Huh7$^{VPS35\text{-}KO}$ cells. HCM images in panel F, examples from a bank of unbiased operator-independent machine-collected and algorithm-processed fields containing a minimum of 500 primary objects/cells per well (5 wells minimum per 96-well plate; 3 plates minimum), per cell line/condition.

bioRxiv preprint doi: https://doi.org/10.1101/2024.07.10.602886; this version posted October 14, 2024. The copyright holder for this preprint (which was not certified by peer review) is the author/funder, who has granted bioRxiv a license to display the preprint in perpetuity. It is made available under aCC-BY 4.0 International license.

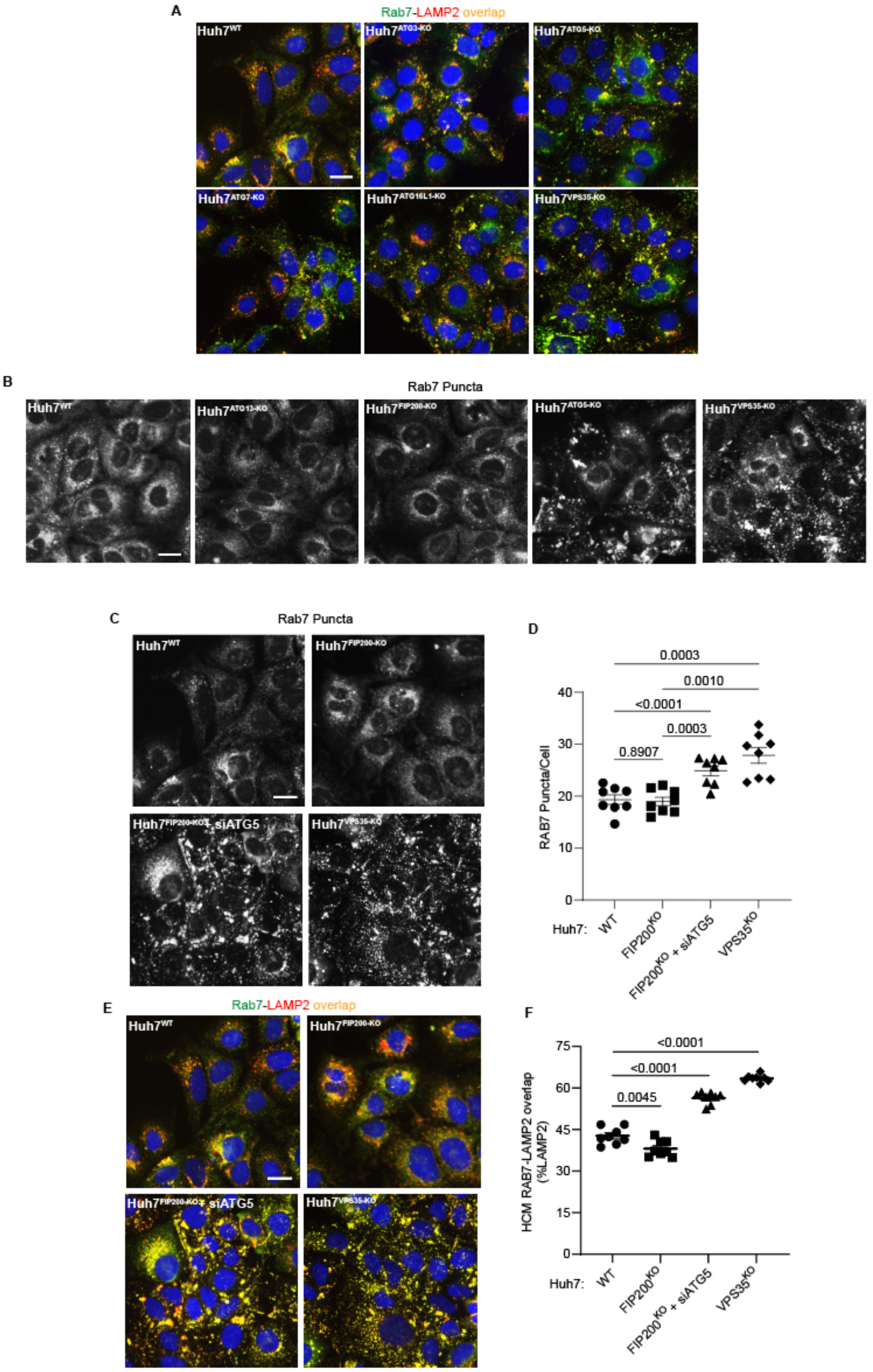

Supplementary Figure S6. Loss of membrane atg8ylation but not of canonical autophagy diverts of RAB7 to lysosomal compartments. A. HCM images of Rab7 (immunostaining of endogenous protein) showing Rab7 colocalization with LAMP2 (% of LAMP2 profiles positive for GLUT1; overlap area) in Huh7^{WT}, Huh7$^{ATG3\text{-}KO}$, Huh7$^{ATG5\text{-}KO}$,

bioRxiv preprint doi: https://doi.org/10.1101/2024.07.10.602886; this version posted October 14, 2024. The copyright holder for this preprint (which was not certified by peer review) is the author/funder, who has granted bioRxiv a license to display the preprint in perpetuity. It is made available under aCC-BY 4.0 International license.

Huh7$^{ATG7\text{-}KO}$, Huh7$^{ATG16\text{-}KO}$, and Huh7$^{VPS35\text{-}KO}$ cells. Scale bar, 20 μm. **B.** HCM images of Rab7 (puncta/cell of endogenous Rab7 profiles stained for immunofluorescence) in Huh7^{WT}, Huh7$^{ATG13\text{-}KO}$, Huh7$^{FIP200\text{-}KO}$, Huh7$^{ATG5\text{-}KO}$, and Huh7$^{VPS35\text{-}KO}$ cells. Scale bar, 20 μm. **C,D.** HCM images (C) and quantification of RAB7 puncta (D) in Huh7^{WT}, Huh7$^{FIP200\text{-}KO}$, Huh7$^{FIP200\text{-}KO\ +\ siATG5}$, and Huh7$^{VPS35\text{-}KO}$ cells. Scale bar, 20 μm. Data, means ± SE (n =8), one-way ANOVA with Tukey's multiple comparisons. **E,F.** HCM images (E) and quantification of RAB7-LAMP2 colocalization (F) in Huh7^{WT}, Huh7$^{FIP200\text{-}KO}$, Huh7$^{FIP200\text{-}KO\ +\ siATG5}$, and Huh7$^{VPS35\text{-}KO}$ cells. Scale bar, 20 μm. Data, means ± SE (n =8), one-way ANOVA with Tukey's multiple comparisons. HCM images in all relevant panels, examples from a bank of unbiased operator-independent machine-collected and algorithm-processed fields containing a minimum of 500 primary objects/cells per well (5 wells minimum per 96-well plate; 3 plates minimum), per cell line.

bioRxiv preprint doi: https://doi.org/10.1101/2024.07.10.602886; this version posted October 14, 2024. The copyright holder for this preprint (which was not certified by peer review) is the author/funder, who has granted bioRxiv a license to display the preprint in perpetuity. It is made available under aCC-BY 4.0 International license.

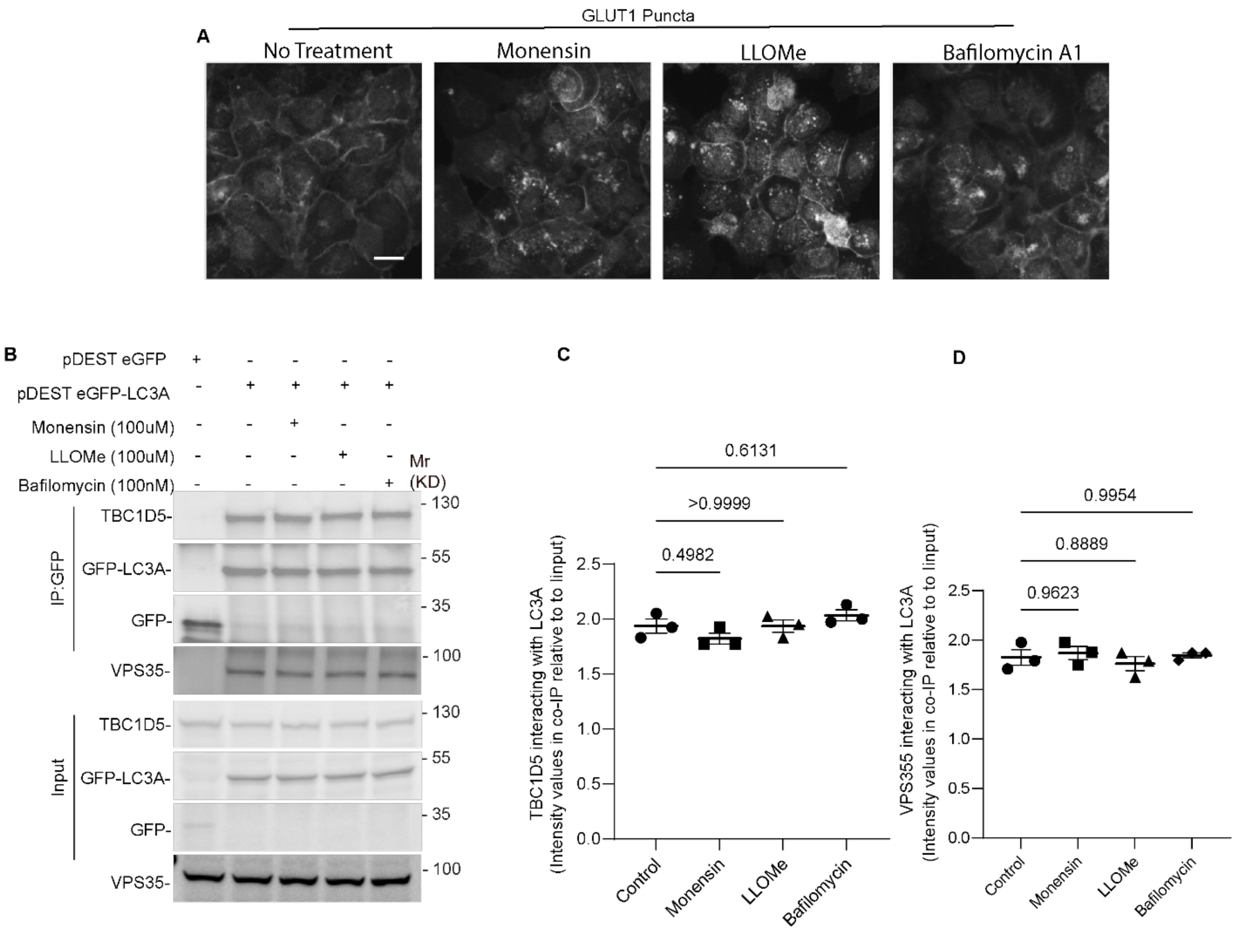

Supplementary Figure S7. CASM agonists effects on retromer cargo GLUT1 in the absence of changes in TBC1D5-LC3A association. A. HCM images of GLUT1 puncta in Huh7^{WT} cells upon treatment with Monensin (100μM), LLOMe (100μM), and Bafilomycin A1 (100nM) for 45 minutes. **B-D.** Co-IP analysis (B) and quantification of TBC1D5 (C) and VPS35 (D) interaction with GFP-LC3 in Huh7 cells treated with or without Monensin (100μM), LLOMe (100μM), and Bafilomycin A1 (100nM) for 45 minutes. Data, means ± SE (n=3), one-way ANOVA with Tukey's multiple comparisons. HCM images in A, examples from a bank of unbiased operator-independent machine-collected and algorithm-processed fields containing a minimum of 500 primary objects/cells per well (5 wells minimum per 96-well plate; 3 plates minimum), per condition.

bioRxiv preprint doi: https://doi.org/10.1101/2024.07.10.602886; this version posted October 14, 2024. The copyright holder for this preprint (which was not certified by peer review) is the author/funder, who has granted bioRxiv a license to display the preprint in perpetuity. It is made available under aCC-BY 4.0 International license.

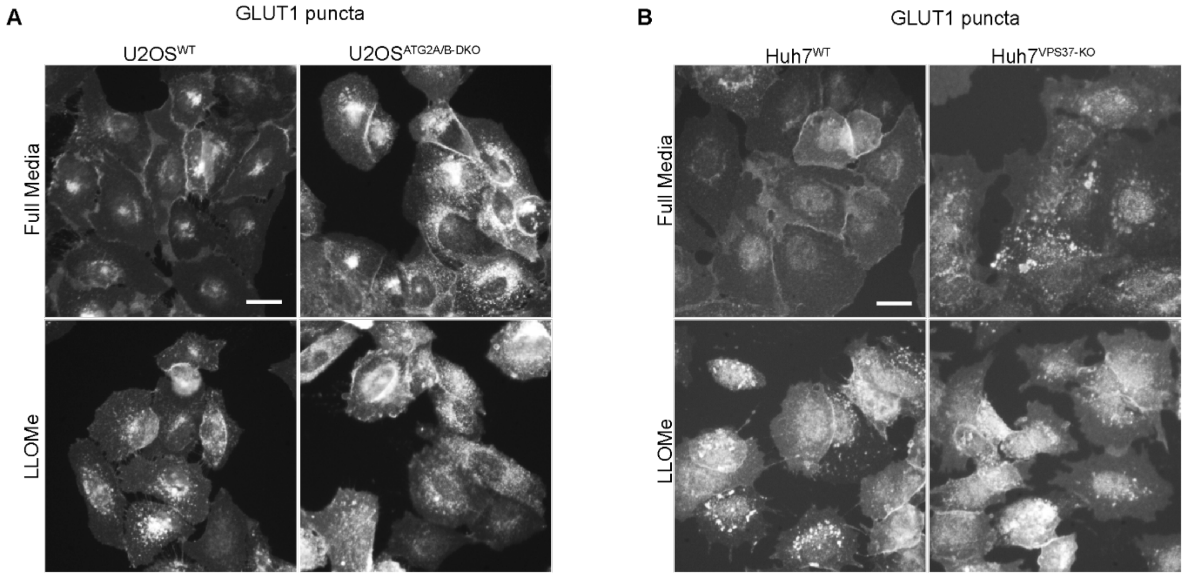

Supplementary Figure S8. HCM images of GLUT1. A. HCM representative images of GLUT1 puncta; immunostaining of endogenous protein in U2OS^{WT} and U2OS$^{ATG2A/B\text{-}DKO}$ cells upon treatment with LLOMe (100µM) for 45 min. **B.** HCM representative images of GLUT1 puncta in Huh7^{WT} and Huh7$^{VPS37A\text{-}KO}$ cells upon treatment with LLOMe (100µM) for 45 min.

bioRxiv preprint doi: https://doi.org/10.1101/2024.07.10.602886; this version posted October 14, 2024. The copyright holder for this preprint (which was not certified by peer review) is the author/funder, who has granted bioRxiv a license to display the preprint in perpetuity. It is made available under aCC-BY 4.0 International license.

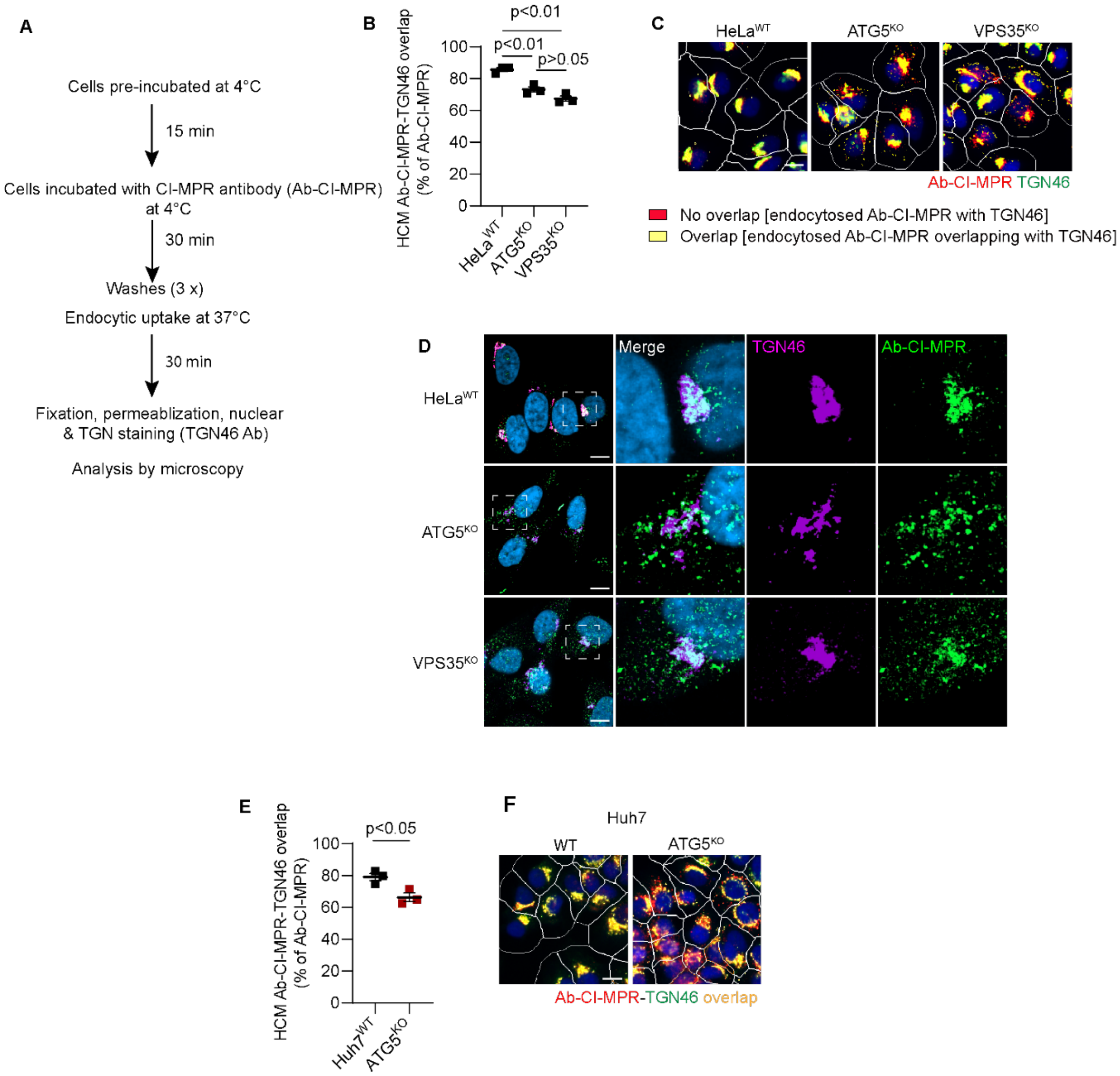

Supplementary Figure S9. Effects of ATG5 knockout on MPR sorting. A. Pulse-chase procedure for monitoring CI-MPR trafficking following CI-MPR antibody uptake. **B,C.** HCM quantification and representative images of Ab-CI-MPR colocalization with TGN46 after 30 min of intracellular sorting in HeLa^{WT}, HeLa$^{ATG5\text{-}KO}$, and HeLa$^{VPS35\text{-}KO}$ cells. Yellow masks represent machine-assigned colocalization of Ab-CI-MPR and TGN46. Data, means ± SE (n=3); one-way ANOVA with Tukey's multiple comparisons. **D.** Confocal images of Ab-CI-MPR antibody localization relative to TGN46 in HeLa^{WT}, HeLa$^{ATG5\text{-}KO}$, and HeLa$^{VPS35\text{-}KO}$ cells. **E, F.** HCM quantification and representative images of Ab-CI-MPR colocalization with TGN46 after 30 min of intracellular sorting in Huh7^{WT} and Huh7$^{ATG5\text{-}KO}$ cells. Data, means ± SE (n=3); unpaired *t*-test. Each experiment

bioRxiv preprint doi: https://doi.org/10.1101/2024.07.10.602886; this version posted October 14, 2024. The copyright holder for this preprint (which was not certified by peer review) is the author/funder, who has granted bioRxiv a license to display the preprint in perpetuity. It is made available under aCC-BY 4.0 International license.

(independent biological repeats; n=3) consists of machine-identified 500 valid primary objects/cells per well, ≥5 wells/sample. All data collection, processing (object, ROI, and target mask assignments) and analyses were computer driven independently of human operators.

bioRxiv preprint doi: https://doi.org/10.1101/2024.07.10.602886; this version posted October 14, 2024. The copyright holder for this preprint (which was not certified by peer review) is the author/funder, who has granted bioRxiv a license to display the preprint in perpetuity. It is made available under aCC-BY 4.0 International license.

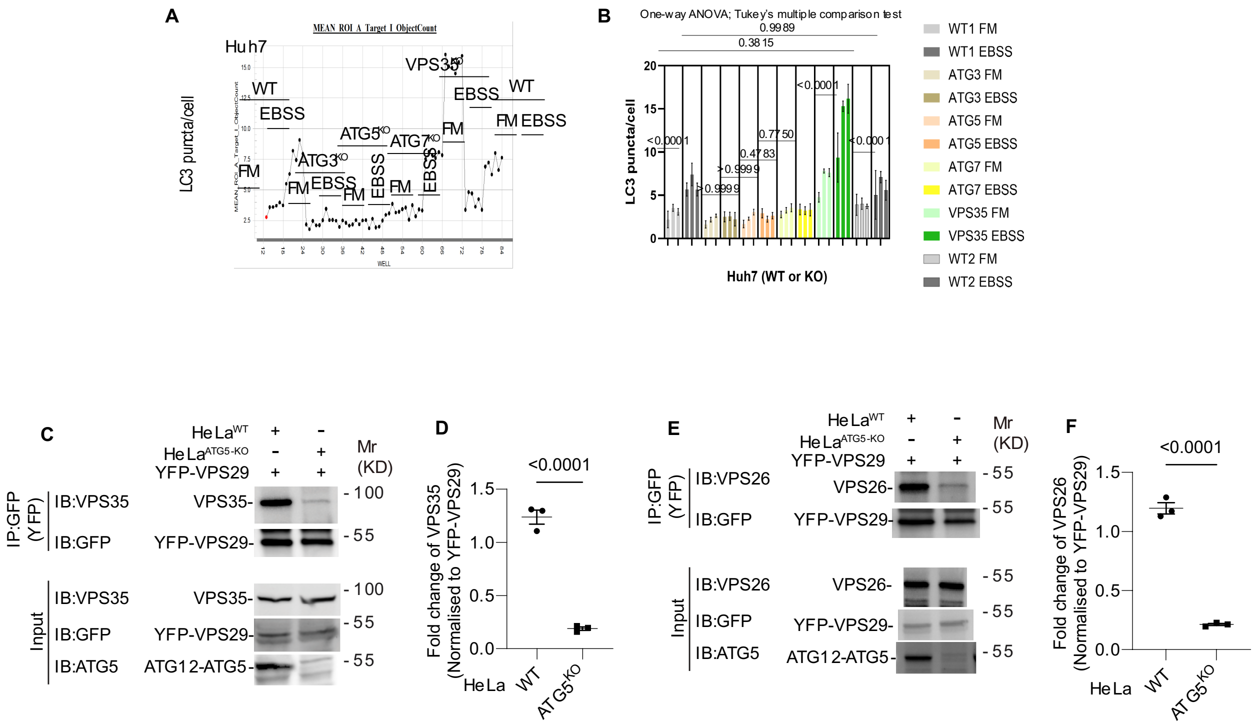

Supplementary Figure S10. ATG5 affects retromer assembly. A,B. HCM quantification of LC3 puncta in Huh7^{WT}, Huh7$^{ATG3\text{-}KO}$, Huh7$^{ATG5\text{-}KO}$, Huh7$^{ATG7\text{-}KO}$, and Huh7$^{VPS35\text{-}KO}$ cells induced for autophagy in EBSS for 90 minutes. Plot in C, an example of a whole 96-well plate HCM readout; X axis, well positions; Y axis, HCM parameter quantified. Plot in D, nested data from three different plates each one as in C. FM, full medium; EVSS, starvation medium. Data, means ± SE (n=3); one-way ANOVA with Tukey's multiple comparisons. **C,D.** Co-IP analysis (E) and quantification (F) of VPS35 and YFP-VPS29 interaction in HeLa^{WT}, and HeLa$^{ATG5\text{-}KO}$ cells. Data, means ± SE (n=3), one-way ANOVA with Tukey's multiple comparisons. **E,F.** Co-IP analysis (G) and quantification (H) of VPS26 and YFP-VPS29 interaction in HeLa^{WT}, and HeLa$^{ATG5\text{-}KO}$ cells. Data, means ± SE (n=3); unpaired *t*-test.
